# Cochrane Skin Group’s Global Social Media Reach: Content Analysis of Facebook, Instagram, and Twitter Posts

**DOI:** 10.2196/40905

**Published:** 2022-11-30

**Authors:** Areebah Ahmad, Lina Alhanshali, Itisha S Jefferson, Robert Dellavalle

**Affiliations:** 1 McGovern Medical School University of Texas Health Science Center Houston, TX United States; 2 SUNY Downstate Health Sciences University College of Medicine Brooklyn, NY United States; 3 Loyola University Stritch School of Medicine Maywood, IL United States; 4 Department of Dermatology University of Colorado School of Medicine Aurora, CO United States

**Keywords:** social media, Cochrane Skin, dermatology, content engagement, Facebook, Cochrane, Twitter, social media analysis, content analysis, skin disease, dermatologist

## Abstract

**Background:**

Researchers in all medical specialties increasingly use social media to educate the public, share new publications with peers, and diversify their audiences.

**Objective:**

Given Cochrane Skin Group’s expanded use of social media in the past years, we aimed to characterize Cochrane Skin Group's international social media audience and identify themes that result in increased content engagement.

**Methods:**

Cochrane Skin Group's Facebook, Instagram, and Twitter analytics data were extracted for follower demographics and the most viewed posts within a 3-year span (June 2019 to June 2022).

**Results:**

Overall, Cochrane Skin Group had the highest number of followers on Facebook (n=1037). The number of Instagram and Twitter followers reached 214 and 352, respectively. The greatest numbers of Facebook followers were from Brazil, Egypt, and India, with 271, 299, and 463 followers, respectively. Facebook’s most viewed post about Cochrane Skin Group’s annual meeting received 1041 views. The top post on Instagram, which introduced Cochrane Skin Group’s social media editors, received 2522 views.

**Conclusions:**

Each of the social media platforms used by Cochrane Skin Group reached varying audiences all over the world. Across social media platforms, posts regarding Cochrane Skin Group meetings, members, and professional opportunities received the most views. Overall, Cochrane Skin Group's multiplatform social media approach will continue to grow an international audience, connecting people interested in skin disease.

## Introduction

The goal of Cochrane Skin Group (CSG) is to publish systematic reviews regarding all aspects of skin disease, including prevention, management, and treatment [1]. CSG has been an international leader in dermatoepidemiology and evidence-based dermatology since its creation 25 years ago [2]. To encourage the dissemination of review information, CSG has used social media to reach a broad global audience. As previously described, social media provides an accessible and popular avenue for the sharing of health care information, networking, and outreach in medicine [3]. In this study, we aimed to characterize CSG’s international social media audience and identify themes that result in increased content engagement.

## Methods

Facebook, Instagram, and Twitter analytics data were extracted for followers’ country of origin and sex as of June 28, 2022. The top 3 countries of origin for followers of each platform were recorded. Each social media platform uses varying terminology to refer to the number of people who have seen a post; Facebook uses “reach,” and Instagram and Twitter use “impressions.” For clarity, we refer to the number of people who have seen a post as “views.” Posts with the highest number of views within a 3-year span (June 2019 to June 2022) were extracted.

## Results

CSG had 1037, 214, and 352 Facebook, Instagram, and Twitter followers, respectively. Among CSG’s Facebook followers, 43.4% (450/1037) were female, and 56.6% (587/1037) were male; 44.6% (463/1037) were from India, 26.2% (271/1037) were from Brazil, and 22.1% (299/1037) were from Egypt (Table 1). Among CSG’s Instagram followers, 52.3% (112/214) were female, and 47.7% (102/214) were male; 25.7% (55/214) were from Brazil, 13.6% (29/214) were from the United States, and 5.1% (11/214) were from Iran (Table 1). Among CSG’s Twitter followers, 35.8% (126/352) were female, and 64.2% (226/352) were male; 28.1% (99/352) were from the United Kingdom, 13.6% (48/352) were from the United States, and 5.4% (19/352) were from Spain (Table 1).

The CSG’s posts with the greatest number of views were all posted within the last year. Facebook’s top post about CSG’s annual meeting at the American Academy of Dermatology Conference received 1041 views (Table 2). The top post on Instagram, which introduced CSG’s social media editors, received 2522 views (Table 2). The top post on Twitter, which highlighted a dermatoepidemiology research fellowship opportunity, received 4422 views.

**Table 1:** Demographics of Cochrane Skin Group Facebook, Instagram, and Twitter followers.

Variable		Facebook	Instagram	Twitter
Followers, n		1037	214	352
**Sex, n (%)**				
	Female	450 (43.4)	112 (52.3)	126 (35.8)
	Male	587 (56.6)	102 (47.7)	226 (64.2)
**Country^a^, n (%)**				
	Brazil	271 (26.2)	55 (25.7)	—^b^
	Egypt	299 (22.1)	—	—
	India	463 (44.6)	—	—
	Iran	—	11 (5.1)	—
	Spain	—	—	19 (5.4)
	Taiwan	40 (3.9)	—	19 (5.4)
	United Kingdom	—	—	99 (28.1)
	United States	—	29 (13.6)	48 (13.6)

^a^The countries listed each have greater than 3% of the social media platform followers.

^b^Not available.

**Table 2:** The highest viewed posts on the social media platforms used by Cochrane Skin Group.

Title of post	Type of post	Date posted	Views, n	Platform
“We hope everyone had a great time at the AAD annual meeting…” [4]	CSG^a^ meeting	August 9, 2021	1041	Facebook
“We would like to introduce ourselves as the new Cochrane Skin’s social media editors…” [5]	CSG editors	July 28, 2021	2522	Instagram
“Dermatoepidemiology Fellowship Opportunity…” [6]	Fellowship opportunity	May 22, 2022	4422	Twitter

^a^CSG: Cochrane Skin Group.

## Discussion

Overall, the countries of origin for followers of CSG social media accounts vary by platform. CSG Facebook followers are predominantly from South Asia and Africa, while Instagram followers are primarily from North America and South America. Furthermore, Twitter followers are primarily from the United States and United Kingdom—the same countries of origin as those of CSG’s coordinating editors. These differences in audience background between each social media platform suggest that CSG’s multiplatform social media approach allows information to be spread to a broader international audience.

Generally, the most viewed social media posts involved content regarding CSG meetings, members, and professional opportunities. Therefore, posting content that references CSG’s mission, events, and team can be prioritized alongside review dissemination to engage established and new followers. Although CSG’s Facebook page had the most followers, CSG’s Twitter posts consistently had a greater number of views, with Twitter’s top post having 3 times the number of views when compared to Facebook’s top post. As described in previous studies, Twitter is the most popular social media platform for health care communication [7], which may explain CSG’s high levels of Twitter engagement.

Some recommendations to help further enhance CSG’s social media presence may include, but are not limited to, (1) creating polls to ask users for their opinion on the most valuable content and (2) embedding social media content into newsletters and blog posts. Although our study is specific to CSG’s social media analytics data from the last 3 years, our highest performing social media posts can act as a guide for other journals interested in expanding their digital reach. Concise posts that are specific to editorial board members and research opportunities tend to accrue the most engagement. As CSG’s social media presence continues to grow, it will provide new ways to connect with an international audience interested in dermatology.

## Abbreviations

CSG: Cochrane Skin Group
